# *catena*-Poly[[triphenyl­tin(IV)]-μ_2_-[3-(cyclo­hexyl­carbamo­yl)propanoato-κ^2^ *O* ^1^:*O* ^3^]]

**DOI:** 10.1107/S1600536810036500

**Published:** 2010-09-18

**Authors:** S. Shams-ul-Islam, Moazzam H. Bhatti, Imtiaz-ud-Din, Seik Weng Ng, Edward R. T. Tiekink

**Affiliations:** a Department of Chemistry, Allama Iqbal Open University, Islamabad 44000, Pakistan; b Department of Chemistry, University of Malaya, 50603 Kuala Lumpur, Malaysia

## Abstract

The Sn atom in the polymeric title compound, [Sn(C_6_H_5_)_3_(C_10_H_16_NO_3_)]_*n*_, is five-coordinated within a *trans*-C_3_O_2_ donor set that defines an approximate trigonal-bipyramidal geometry. The carboxyl­ate ligand is monodentate and the amide O atom bridges a symmetry-related Sn atom, generating a chain along [010] with a linear topology. An intra­molecular carboxyl­ate–carbonyl N—H⋯O hydrogen bond is responsible for the curved conformation within the carboxyl­ate ligand.

## Related literature

For reviews of organotin carboxyl­ate structures, see: Ng *et al.* (1986 ▶); Tiekink (1991 ▶). For the influence of steric effects upon structural motifs, see: Willem *et al.* (1998 ▶). For a closely related structure, see: Imtiaz-ud-Din *et al.* (2010 ▶). For additional geometric analysis, see: Addison *et al.* (1984 ▶). For the synthesis of *N*-cyclo­hexyl­succinamic acid, see: Dolzhenko *et al.* (2003 ▶).
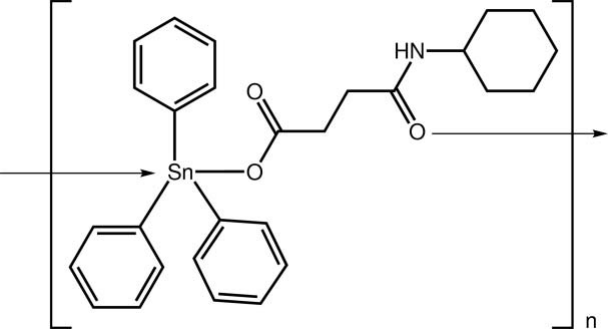


## Experimental

### 

#### Crystal data

[Sn(C_6_H_5_)_3_(C_10_H_16_NO_3_)]
*M* *_r_* = 548.23
Monoclinic, 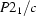

*a* = 16.2488 (12) Å
*b* = 9.1243 (7) Å
*c* = 17.6597 (13) Å
β = 106.101 (1)°
*V* = 2515.5 (3) Å^3^
*Z* = 4
Mo *K*α radiation
μ = 1.04 mm^−1^
*T* = 100 K
0.25 × 0.15 × 0.05 mm

#### Data collection

Bruker SMART APEX diffractometer
Absorption correction: multi-scan (*SADABS*; Sheldrick, 1996 ▶) *T*_min_ = 0.634, *T*_max_ = 0.746
23281 measured reflections
5756 independent reflections
4864 reflections with *I* > 2σ(*I*)
*R*_int_ = 0.047

#### Refinement

*R*[*F* ^2^ > 2σ(*F* ^2^)] = 0.028
*wR*(*F* ^2^) = 0.084
*S* = 1.03
5756 reflections
302 parameters
1 restraint
H atoms treated by a mixture of independent and constrained refinement
Δρ_max_ = 0.53 e Å^−3^
Δρ_min_ = −0.64 e Å^−3^

### 

Data collection: *APEX2* (Bruker, 2009 ▶); cell refinement: *SAINT* (Bruker, 2009 ▶); data reduction: *SAINT*; program(s) used to solve structure: *SHELXS97* (Sheldrick, 2008 ▶); program(s) used to refine structure: *SHELXL97* (Sheldrick, 2008 ▶); molecular graphics: *ORTEP-3* (Farrugia, 1997 ▶) and *DIAMOND* (Brandenburg, 2006 ▶); software used to prepare material for publication: *publCIF* (Westrip, 2010 ▶).

## Supplementary Material

Crystal structure: contains datablocks global, I. DOI: 10.1107/S1600536810036500/hg2713sup1.cif

Structure factors: contains datablocks I. DOI: 10.1107/S1600536810036500/hg2713Isup2.hkl

Additional supplementary materials:  crystallographic information; 3D view; checkCIF report

## Figures and Tables

**Table 1:** Selected bond lengths (Å)

Sn—O1	2.1658 (17)
Sn—O3^i^	2.3178 (16)
Sn—C1	2.138 (2)
Sn—C7	2.129 (3)
Sn—C13	2.130 (3)

Symmetry code: (i) 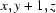.

**Table 2:** Hydrogen-bond geometry (Å, °)

*D*—H⋯*A*	*D*—H	H⋯*A*	*D*⋯*A*	*D*—H⋯*A*
N1—H1⋯O2	0.86 (1)	1.93 (2)	2.732 (3)	155 (3)

**Fig. 1.**
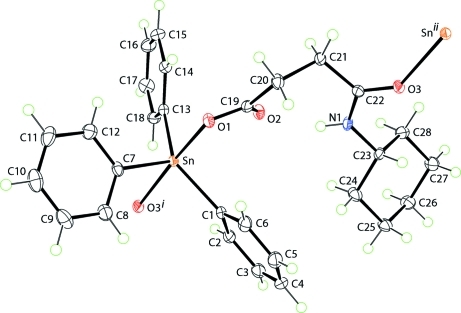
The molecular structure of the asymmetric unit in (I) extended to show the bridging atoms and showing the atom-labelling scheme with displacement ellipsoids at the 50% probability level. Symmetry operation i: x, 1 + y, z; ii: x, -1 + y, z.

**Fig. 2.**
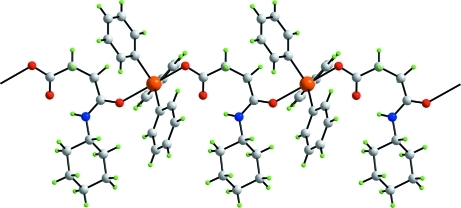
A portion of the linear polymeric chain in (I). Colour code: Sn, orange; O, red; N, blue; C, grey; H, green.

## supplementary crystallographic information

### Comment

Triorganotin carboxylates are usually monomeric or polymeric (Ng *et al.*, 1986; Tiekink, 1991), depending largely on steric factors (Willem *et al.*, 1998). As a continuation of recent studies on triorganotin carboxylates having carboxylate ligands with additional coordination functionality (Imtiaz-ud-Din *et al.*, 2010), the title compound, (I), was investigated.

In (I), Fig. 1, the Sn atom is coordinated by three *ipso*-C atoms derived from three phenyl groups, an O atom from a monodentate carboxylate ligand, and, from a symmetry related molecule, an amide-O atom, to define a *trans*-C_3_O_2_ coordination geometry, Table 1. The value of τ computes to 0.73 compared to τ = 1.0 for an ideal trigonal bipyramid and τ = 0.0 for an ideal square pyramid (Addison *et al.*, 1984). The distortion in the Sn atom geometry is ascribed, in part, to the close approach of the O2 atom; Sn···O2 = 2.9936 (17) Å. The central part of the carboxylate ligand is curved, *e.g*. the C19–C20–C21–C22 torsion angle = -77.3 (3) °, owing to the presence of an intramolecular N–H···O2 hydrogen bond, Table 2. The resulting supramolecular chain has a linear topology, Fig. 2, in contrast to the helical chain found recently in the structure of the benzoate derivative (Imtiaz-ud-Din *et al.*, 2010).

### Experimental

*N*-Cyclohexylsuccinamic acid was synthesized from the reaction of succinic anhydride and cyclohexylamine in ethyl acetate by the methodology reported earlier (Dolzhenko *et al.*, 2003). Triphenyltin hydroxide (3.17 g, 10 mmol) and *N*-cyclohexylsuccinamic acid (2.66 g, 10 mmol) were heated to reflux in 50 ml dry ethanol/acetone mixture (8:2) for 6–8 h, The solvent was then removed under reduced pressure and the solid mass thus obtained was recrystallized from a mixture of chloroform and n-hexane (3:1) to furnish colourless crystals.

### Refinement

Carbon-bound H-atoms were placed in calculated positions (C—H 0.93 to 0.96 Å) and were included in the refinement in the riding model approximation, with *U*_iso_(H) set to 1.2 to 1.5*U*_equiv_(C). The N-bound H-atom was located in a difference Fourier map, and was refined with a distance restraint of N—H 0.86±0.01 Å; the *U_iso_* value was freely refined.

### Crystal data

**Table:**

[Sn(C_6_H_5_)_3_(C_10_H_16_NO_3_)]	*F*(000) = 1120
*M**_r_* = 548.23	*D*_x_ = 1.448 Mg m^−^^3^
Monoclinic, *P*2_1_/*c*	Mo *K*α radiation, λ = 0.71073 Å
Hall symbol: -P 2ybc	Cell parameters from 5969 reflections
*a* = 16.2488 (12) Å	θ = 2.4–28.2°
*b* = 9.1243 (7) Å	µ = 1.04 mm^−^^1^
*c* = 17.6597 (13) Å	*T* = 100 K
β = 106.101 (1)°	Block, colourless
*V* = 2515.5 (3) Å^3^	0.25 × 0.15 × 0.05 mm
*Z* = 4	

### Data collection

**Table:**

Bruker SMART APEX diffractometer	5756 independent reflections
Radiation source: fine-focus sealed tube	4864 reflections with *I* > 2σ(*I*)
graphite	*R*_int_ = 0.047
ω scan	θ_max_ = 27.5°, θ_min_ = 2.4°
Absorption correction: multi-scan (*SADABS*; Sheldrick, 1996)	*h* = −21→21
*T*_min_ = 0.634, *T*_max_ = 0.746	*k* = −11→11
23281 measured reflections	*l* = −22→22

### Refinement

**Table:**

Refinement on *F*^2^	Primary atom site location: structure-invariant direct methods
Least-squares matrix: full	Secondary atom site location: difference Fourier map
*R*[*F*^2^ > 2σ(*F*^2^)] = 0.028	Hydrogen site location: inferred from neighbouring sites
*wR*(*F*^2^) = 0.084	H atoms treated by a mixture of independent and constrained refinement
*S* = 1.03	*w* = 1/[σ^2^(*F*_o_^2^) + (0.0481*P*)^2^] where *P* = (*F*_o_^2^ + 2*F*_c_^2^)/3
5756 reflections	(Δ/σ)_max_ = 0.002
302 parameters	Δρ_max_ = 0.53 e Å^−^^3^
1 restraint	Δρ_min_ = −0.64 e Å^−^^3^

### Special details

**Table:**

Geometry. All e.s.d.'s (except the e.s.d. in the dihedral angle between two l.s. planes) are estimated using the full covariance matrix. The cell e.s.d.'s are taken into account individually in the estimation of e.s.d.'s in distances, angles and torsion angles; correlations between e.s.d.'s in cell parameters are only used when they are defined by crystal symmetry. An approximate (isotropic) treatment of cell e.s.d.'s is used for estimating e.s.d.'s involving l.s. planes.
Refinement. Refinement of *F*^2^ against ALL reflections. The weighted *R*-factor *wR* and goodness of fit *S* are based on *F*^2^, conventional *R*-factors *R* are based on *F*, with *F* set to zero for negative *F*^2^. The threshold expression of *F*^2^ > σ(*F*^2^) is used only for calculating *R*-factors(gt) *etc*. and is not relevant to the choice of reflections for refinement. *R*-factors based on *F*^2^ are statistically about twice as large as those based on *F*, and *R*- factors based on ALL data will be even larger.

### Fractional atomic coordinates and isotropic or equivalent isotropic displacement parameters (Å^2^)

**Table:**

	*x*	*y*	*z*	*U*_iso_*/*U*_eq_	
Sn	0.713719 (10)	0.684277 (17)	0.636309 (10)	0.01166 (7)	
O1	0.69004 (11)	0.47709 (18)	0.57420 (10)	0.0155 (4)	
O2	0.67770 (12)	0.38360 (18)	0.68702 (10)	0.0167 (4)	
O3	0.75097 (11)	−0.09345 (18)	0.70015 (10)	0.0162 (4)	
N1	0.76074 (14)	0.1326 (2)	0.74976 (12)	0.0155 (4)	
H1	0.7445 (18)	0.2220 (14)	0.7429 (17)	0.021 (8)*	
C1	0.83515 (16)	0.6177 (3)	0.71146 (15)	0.0135 (5)	
C2	0.86804 (18)	0.6706 (3)	0.78784 (16)	0.0182 (6)	
H2	0.8346	0.7352	0.8094	0.022*	
C3	0.94956 (19)	0.6294 (3)	0.83297 (18)	0.0260 (6)	
H3	0.9713	0.6659	0.8851	0.031*	
C4	0.99895 (18)	0.5356 (3)	0.80199 (18)	0.0272 (7)	
H4	1.0549	0.5090	0.8323	0.033*	
C5	0.96632 (18)	0.4809 (3)	0.72680 (18)	0.0249 (6)	
H5	0.9998	0.4160	0.7055	0.030*	
C6	0.88493 (17)	0.5203 (3)	0.68220 (16)	0.0196 (6)	
H6	0.8627	0.4803	0.6309	0.023*	
C7	0.71739 (17)	0.7899 (3)	0.52981 (15)	0.0156 (5)	
C8	0.79231 (19)	0.8554 (3)	0.52171 (17)	0.0212 (6)	
H8	0.8436	0.8495	0.5636	0.025*	
C9	0.7924 (2)	0.9291 (3)	0.45295 (18)	0.0283 (7)	
H9	0.8441	0.9712	0.4479	0.034*	
C10	0.7182 (2)	0.9418 (3)	0.39173 (17)	0.0286 (7)	
H10	0.7187	0.9930	0.3450	0.034*	
C11	0.6436 (2)	0.8797 (3)	0.39911 (17)	0.0275 (7)	
H11	0.5922	0.8888	0.3575	0.033*	
C12	0.64319 (19)	0.8035 (3)	0.46733 (17)	0.0221 (6)	
H12	0.5915	0.7601	0.4714	0.026*	
C13	0.59173 (17)	0.7011 (3)	0.65848 (15)	0.0149 (5)	
C14	0.52338 (17)	0.6196 (3)	0.61153 (16)	0.0177 (5)	
H14	0.5331	0.5557	0.5724	0.021*	
C15	0.44184 (17)	0.6309 (3)	0.62125 (17)	0.0216 (6)	
H15	0.3962	0.5747	0.5891	0.026*	
C16	0.42711 (18)	0.7244 (3)	0.67808 (18)	0.0240 (6)	
H16	0.3713	0.7320	0.6848	0.029*	
C17	0.49320 (19)	0.8064 (3)	0.72480 (18)	0.0235 (6)	
H17	0.4829	0.8708	0.7634	0.028*	
C18	0.57512 (17)	0.7944 (3)	0.71517 (16)	0.0174 (5)	
H18	0.6205	0.8507	0.7477	0.021*	
C19	0.68113 (16)	0.3687 (3)	0.61824 (15)	0.0144 (5)	
C20	0.67628 (18)	0.2192 (3)	0.57931 (16)	0.0183 (6)	
H20A	0.7324	0.1979	0.5700	0.022*	
H20B	0.6332	0.2238	0.5273	0.022*	
C21	0.65302 (16)	0.0911 (3)	0.62635 (15)	0.0164 (5)	
H21A	0.6050	0.1222	0.6470	0.020*	
H21B	0.6324	0.0082	0.5898	0.020*	
C22	0.72532 (16)	0.0370 (3)	0.69461 (15)	0.0141 (5)	
C23	0.82697 (16)	0.0973 (3)	0.82208 (14)	0.0141 (5)	
H23	0.8668	0.0238	0.8094	0.017*	
C24	0.87745 (18)	0.2358 (3)	0.85281 (15)	0.0211 (6)	
H24A	0.9053	0.2719	0.8132	0.025*	
H24B	0.8377	0.3127	0.8608	0.025*	
C25	0.94531 (18)	0.2078 (3)	0.93013 (16)	0.0205 (6)	
H25A	0.9733	0.3016	0.9508	0.025*	
H25B	0.9897	0.1416	0.9206	0.025*	
C26	0.90676 (18)	0.1391 (3)	0.99144 (16)	0.0205 (6)	
H26A	0.8679	0.2104	1.0062	0.025*	
H26B	0.9532	0.1151	1.0394	0.025*	
C27	0.85695 (18)	−0.0001 (3)	0.95937 (15)	0.0209 (6)	
H27A	0.8968	−0.0749	0.9495	0.025*	
H27B	0.8302	−0.0398	0.9990	0.025*	
C28	0.78805 (16)	0.0314 (3)	0.88344 (15)	0.0181 (5)	
H28A	0.7456	0.1005	0.8942	0.022*	
H28B	0.7579	−0.0606	0.8628	0.022*	

### Atomic displacement parameters (Å^2^)

**Table:**

	*U*^11^	*U*^22^	*U*^33^	*U*^12^	*U*^13^	*U*^23^
Sn	0.01279 (11)	0.00991 (10)	0.01152 (10)	0.00003 (6)	0.00211 (7)	0.00066 (6)
O1	0.0210 (10)	0.0116 (8)	0.0127 (9)	−0.0004 (7)	0.0027 (8)	0.0005 (7)
O2	0.0217 (10)	0.0125 (8)	0.0159 (10)	0.0022 (7)	0.0054 (8)	−0.0001 (7)
O3	0.0180 (10)	0.0100 (8)	0.0173 (9)	0.0000 (7)	−0.0006 (8)	−0.0013 (7)
N1	0.0185 (12)	0.0107 (10)	0.0141 (11)	0.0035 (8)	−0.0005 (9)	0.0009 (8)
C1	0.0144 (13)	0.0116 (11)	0.0142 (12)	0.0001 (9)	0.0033 (10)	0.0034 (9)
C2	0.0194 (14)	0.0130 (12)	0.0199 (14)	−0.0009 (10)	0.0016 (12)	−0.0001 (10)
C3	0.0270 (16)	0.0184 (13)	0.0247 (16)	−0.0065 (12)	−0.0059 (13)	0.0014 (12)
C4	0.0130 (14)	0.0244 (14)	0.0386 (18)	−0.0020 (11)	−0.0021 (13)	0.0127 (13)
C5	0.0193 (15)	0.0237 (14)	0.0355 (17)	0.0050 (11)	0.0141 (13)	0.0087 (12)
C6	0.0219 (14)	0.0209 (13)	0.0174 (14)	0.0021 (11)	0.0080 (12)	0.0041 (10)
C7	0.0222 (14)	0.0103 (11)	0.0133 (13)	−0.0007 (10)	0.0035 (11)	−0.0002 (9)
C8	0.0249 (15)	0.0187 (13)	0.0197 (14)	−0.0012 (11)	0.0055 (12)	0.0011 (11)
C9	0.0340 (17)	0.0231 (15)	0.0334 (18)	−0.0037 (12)	0.0184 (15)	0.0056 (12)
C10	0.0446 (19)	0.0237 (15)	0.0207 (15)	0.0027 (13)	0.0142 (14)	0.0055 (12)
C11	0.0346 (18)	0.0283 (15)	0.0155 (14)	0.0050 (13)	0.0000 (13)	0.0046 (12)
C12	0.0217 (15)	0.0250 (15)	0.0184 (14)	−0.0017 (11)	0.0037 (12)	0.0030 (11)
C13	0.0148 (13)	0.0130 (12)	0.0161 (13)	0.0002 (9)	0.0029 (11)	0.0039 (9)
C14	0.0168 (14)	0.0164 (12)	0.0181 (13)	−0.0002 (10)	0.0017 (11)	0.0005 (10)
C15	0.0145 (14)	0.0231 (14)	0.0238 (15)	−0.0023 (11)	−0.0005 (12)	0.0012 (11)
C16	0.0155 (14)	0.0279 (15)	0.0293 (16)	0.0024 (11)	0.0074 (13)	0.0027 (12)
C17	0.0250 (16)	0.0251 (15)	0.0228 (15)	0.0000 (11)	0.0105 (13)	−0.0028 (11)
C18	0.0161 (14)	0.0185 (13)	0.0167 (13)	−0.0026 (10)	0.0030 (11)	−0.0001 (10)
C19	0.0135 (13)	0.0123 (11)	0.0152 (13)	0.0004 (10)	0.0004 (10)	−0.0012 (10)
C20	0.0253 (15)	0.0131 (12)	0.0139 (13)	0.0005 (10)	0.0011 (11)	−0.0006 (10)
C21	0.0175 (13)	0.0107 (11)	0.0173 (13)	0.0002 (9)	−0.0014 (11)	−0.0003 (9)
C22	0.0130 (12)	0.0133 (11)	0.0163 (13)	−0.0010 (9)	0.0049 (10)	0.0015 (10)
C23	0.0145 (13)	0.0119 (11)	0.0134 (12)	0.0013 (9)	−0.0005 (10)	−0.0005 (9)
C24	0.0260 (15)	0.0170 (13)	0.0166 (14)	−0.0061 (11)	−0.0004 (12)	0.0000 (11)
C25	0.0220 (15)	0.0194 (13)	0.0163 (14)	−0.0064 (11)	−0.0006 (12)	0.0003 (10)
C26	0.0220 (15)	0.0230 (13)	0.0149 (13)	−0.0024 (11)	0.0024 (11)	−0.0006 (11)
C27	0.0229 (15)	0.0229 (13)	0.0163 (14)	−0.0042 (11)	0.0041 (12)	0.0044 (11)
C28	0.0154 (14)	0.0192 (13)	0.0190 (14)	−0.0008 (10)	0.0037 (11)	−0.0014 (10)

### Geometric parameters (Å, °)

**Table:**

Sn—O1	2.1658 (17)	C13—C14	1.401 (4)
Sn—O3^i^	2.3178 (16)	C14—C15	1.386 (4)
Sn—C1	2.138 (2)	C14—H14	0.9500
Sn—C7	2.129 (3)	C15—C16	1.388 (4)
Sn—C13	2.130 (3)	C15—H15	0.9500
O1—C19	1.291 (3)	C16—C17	1.379 (4)
O2—C19	1.239 (3)	C16—H16	0.9500
O3—C22	1.256 (3)	C17—C18	1.392 (4)
O3—Sn^ii^	2.3178 (16)	C17—H17	0.9500
N1—C22	1.316 (3)	C18—H18	0.9500
N1—C23	1.460 (3)	C19—C20	1.520 (3)
N1—H1	0.855 (10)	C20—C21	1.540 (3)
C1—C2	1.392 (4)	C20—H20A	0.9900
C1—C6	1.394 (3)	C20—H20B	0.9900
C2—C3	1.395 (4)	C21—C22	1.514 (3)
C2—H2	0.9500	C21—H21A	0.9900
C3—C4	1.386 (4)	C21—H21B	0.9900
C3—H3	0.9500	C23—C28	1.522 (3)
C4—C5	1.379 (4)	C23—C24	1.522 (3)
C4—H4	0.9500	C23—H23	1.0000
C5—C6	1.386 (4)	C24—C25	1.520 (4)
C5—H5	0.9500	C24—H24A	0.9900
C6—H6	0.9500	C24—H24B	0.9900
C7—C12	1.396 (4)	C25—C26	1.528 (4)
C7—C8	1.399 (4)	C25—H25A	0.9900
C8—C9	1.388 (4)	C25—H25B	0.9900
C8—H8	0.9500	C26—C27	1.528 (4)
C9—C10	1.384 (4)	C26—H26A	0.9900
C9—H9	0.9500	C26—H26B	0.9900
C10—C11	1.377 (4)	C27—C28	1.517 (4)
C10—H10	0.9500	C27—H27A	0.9900
C11—C12	1.392 (4)	C27—H27B	0.9900
C11—H11	0.9500	C28—H28A	0.9900
C12—H12	0.9500	C28—H28B	0.9900
C13—C18	1.396 (4)		
			
C13—Sn—C7	112.66 (10)	C15—C16—H16	119.9
C13—Sn—C1	130.70 (10)	C16—C17—C18	119.8 (3)
C7—Sn—C1	115.46 (10)	C16—C17—H17	120.1
C13—Sn—O1	96.45 (8)	C18—C17—H17	120.1
C7—Sn—O1	89.56 (8)	C17—C18—C13	121.1 (3)
C1—Sn—O1	94.03 (8)	C17—C18—H18	119.4
C13—Sn—O3^i^	88.75 (8)	C13—C18—H18	119.4
C7—Sn—O3^i^	88.06 (8)	O2—C19—O1	123.4 (2)
C1—Sn—O3^i^	82.82 (8)	O2—C19—C20	122.1 (2)
O1—Sn—O3^i^	174.78 (6)	O1—C19—C20	114.6 (2)
C19—O1—Sn	113.42 (15)	C19—C20—C21	115.3 (2)
C22—O3—Sn^ii^	139.03 (17)	C19—C20—H20A	108.5
C22—N1—C23	124.5 (2)	C21—C20—H20A	108.5
C22—N1—H1	118 (2)	C19—C20—H20B	108.5
C23—N1—H1	117 (2)	C21—C20—H20B	108.5
C2—C1—C6	118.3 (2)	H20A—C20—H20B	107.5
C2—C1—Sn	122.91 (19)	C22—C21—C20	115.1 (2)
C6—C1—Sn	118.78 (19)	C22—C21—H21A	108.5
C1—C2—C3	120.6 (3)	C20—C21—H21A	108.5
C1—C2—H2	119.7	C22—C21—H21B	108.5
C3—C2—H2	119.7	C20—C21—H21B	108.5
C4—C3—C2	120.2 (3)	H21A—C21—H21B	107.5
C4—C3—H3	119.9	O3—C22—N1	120.2 (2)
C2—C3—H3	119.9	O3—C22—C21	122.6 (2)
C5—C4—C3	119.6 (3)	N1—C22—C21	117.2 (2)
C5—C4—H4	120.2	N1—C23—C28	111.0 (2)
C3—C4—H4	120.2	N1—C23—C24	108.8 (2)
C4—C5—C6	120.3 (3)	C28—C23—C24	111.3 (2)
C4—C5—H5	119.8	N1—C23—H23	108.6
C6—C5—H5	119.8	C28—C23—H23	108.6
C5—C6—C1	121.0 (3)	C24—C23—H23	108.6
C5—C6—H6	119.5	C25—C24—C23	111.5 (2)
C1—C6—H6	119.5	C25—C24—H24A	109.3
C12—C7—C8	117.8 (2)	C23—C24—H24A	109.3
C12—C7—Sn	120.59 (19)	C25—C24—H24B	109.3
C8—C7—Sn	121.5 (2)	C23—C24—H24B	109.3
C9—C8—C7	120.6 (3)	H24A—C24—H24B	108.0
C9—C8—H8	119.7	C24—C25—C26	111.6 (2)
C7—C8—H8	119.7	C24—C25—H25A	109.3
C10—C9—C8	120.8 (3)	C26—C25—H25A	109.3
C10—C9—H9	119.6	C24—C25—H25B	109.3
C8—C9—H9	119.6	C26—C25—H25B	109.3
C11—C10—C9	119.4 (3)	H25A—C25—H25B	108.0
C11—C10—H10	120.3	C25—C26—C27	110.9 (2)
C9—C10—H10	120.3	C25—C26—H26A	109.4
C10—C11—C12	120.2 (3)	C27—C26—H26A	109.4
C10—C11—H11	119.9	C25—C26—H26B	109.4
C12—C11—H11	119.9	C27—C26—H26B	109.4
C11—C12—C7	121.2 (3)	H26A—C26—H26B	108.0
C11—C12—H12	119.4	C28—C27—C26	110.8 (2)
C7—C12—H12	119.4	C28—C27—H27A	109.5
C18—C13—C14	118.0 (2)	C26—C27—H27A	109.5
C18—C13—Sn	123.41 (19)	C28—C27—H27B	109.5
C14—C13—Sn	118.57 (19)	C26—C27—H27B	109.5
C15—C14—C13	121.0 (3)	H27A—C27—H27B	108.1
C15—C14—H14	119.5	C27—C28—C23	110.7 (2)
C13—C14—H14	119.5	C27—C28—H28A	109.5
C14—C15—C16	119.8 (3)	C23—C28—H28A	109.5
C14—C15—H15	120.1	C27—C28—H28B	109.5
C16—C15—H15	120.1	C23—C28—H28B	109.5
C17—C16—C15	120.3 (3)	H28A—C28—H28B	108.1
C17—C16—H16	119.9		
			
C13—Sn—O1—C19	−66.35 (18)	C1—Sn—C13—C18	61.5 (2)
C7—Sn—O1—C19	−179.11 (18)	O1—Sn—C13—C18	162.4 (2)
C1—Sn—O1—C19	65.40 (18)	O3^i^—Sn—C13—C18	−18.0 (2)
O3^i^—Sn—O1—C19	118.1 (7)	C7—Sn—C13—C14	71.7 (2)
C13—Sn—C1—C2	−47.4 (2)	C1—Sn—C13—C14	−121.5 (2)
C7—Sn—C1—C2	119.1 (2)	O1—Sn—C13—C14	−20.6 (2)
O1—Sn—C1—C2	−149.5 (2)	O3^i^—Sn—C13—C14	159.04 (19)
O3^i^—Sn—C1—C2	34.7 (2)	C18—C13—C14—C15	−0.2 (4)
C13—Sn—C1—C6	134.47 (19)	Sn—C13—C14—C15	−177.4 (2)
C7—Sn—C1—C6	−59.0 (2)	C13—C14—C15—C16	0.2 (4)
O1—Sn—C1—C6	32.4 (2)	C14—C15—C16—C17	0.1 (4)
O3^i^—Sn—C1—C6	−143.4 (2)	C15—C16—C17—C18	−0.4 (4)
C6—C1—C2—C3	1.5 (4)	C16—C17—C18—C13	0.4 (4)
Sn—C1—C2—C3	−176.64 (19)	C14—C13—C18—C17	−0.1 (4)
C1—C2—C3—C4	0.2 (4)	Sn—C13—C18—C17	176.9 (2)
C2—C3—C4—C5	−1.2 (4)	Sn—O1—C19—O2	6.9 (3)
C3—C4—C5—C6	0.5 (4)	Sn—O1—C19—C20	−172.26 (16)
C4—C5—C6—C1	1.3 (4)	O2—C19—C20—C21	9.0 (4)
C2—C1—C6—C5	−2.3 (4)	O1—C19—C20—C21	−171.8 (2)
Sn—C1—C6—C5	175.95 (19)	C19—C20—C21—C22	−77.3 (3)
C13—Sn—C7—C12	−28.0 (2)	Sn^ii^—O3—C22—N1	172.88 (17)
C1—Sn—C7—C12	163.02 (19)	Sn^ii^—O3—C22—C21	−6.3 (4)
O1—Sn—C7—C12	68.8 (2)	C23—N1—C22—O3	−3.6 (4)
O3^i^—Sn—C7—C12	−115.9 (2)	C23—N1—C22—C21	175.7 (2)
C13—Sn—C7—C8	148.0 (2)	C20—C21—C22—O3	−122.2 (3)
C1—Sn—C7—C8	−20.9 (2)	C20—C21—C22—N1	58.5 (3)
O1—Sn—C7—C8	−115.2 (2)	C22—N1—C23—C28	−81.0 (3)
O3^i^—Sn—C7—C8	60.2 (2)	C22—N1—C23—C24	156.2 (2)
C12—C7—C8—C9	−1.1 (4)	N1—C23—C24—C25	177.6 (2)
Sn—C7—C8—C9	−177.3 (2)	C28—C23—C24—C25	55.0 (3)
C7—C8—C9—C10	1.4 (4)	C23—C24—C25—C26	−54.0 (3)
C8—C9—C10—C11	−0.5 (4)	C24—C25—C26—C27	54.6 (3)
C9—C10—C11—C12	−0.6 (4)	C25—C26—C27—C28	−56.3 (3)
C10—C11—C12—C7	0.8 (4)	C26—C27—C28—C23	57.3 (3)
C8—C7—C12—C11	0.1 (4)	N1—C23—C28—C27	−178.0 (2)
Sn—C7—C12—C11	176.3 (2)	C24—C23—C28—C27	−56.7 (3)
C7—Sn—C13—C18	−105.3 (2)		

Symmetry codes: (i) *x*, *y*+1, *z*; (ii) *x*, *y*−1, *z*.

### Hydrogen-bond geometry (Å, °)

**Table:**

*D*—H···*A*	*D*—H	H···*A*	*D*···*A*	*D*—H···*A*
N1—H1···O2	0.86 (1)	1.93 (2)	2.732 (3)	155 (3)
